# Holotomography-Based, Label-Free Quantification of Cellular Dry Mass as a Biophysical Indicator of Microglial Aβ Phagocytosis during Senescence

**DOI:** 10.21203/rs.3.rs-8690474/v1

**Published:** 2026-02-02

**Authors:** Sunjeet Saha, Amarnath Singam, Melanie White, Colbey Kimbel, Nikita Gopakumar, Jeong Hee Kim, Jingchun Chen, Ashkan Salamat, Seungman Park

**Affiliations:** University of Nevada, Las Vegas; University of Nevada, Las Vegas; University of Nevada, Las Vegas; University of Nevada, Las Vegas; University of Nevada, Las Vegas; Iowa State University; University of Nevada, Las Vegas; University of Nevada, Las Vegas; University of Nevada, Las Vegas

**Keywords:** microglial cell, holotomography, senescence, phagocytosis, amyloid-β (Aβ), dry mass

## Abstract

Senescent microglia undergo significant molecular and biochemical changes associated with impaired phagocytosis of amyloid-β (Aβ), a process implicated in neurodegenerative disease progression. However, quantitative biophysical metrics capable of capturing this functional impairment—and thus elucidating the role of microglial dysfunction in disease progression—remain limited. In this study, we identify cellular dry mass, measured by label-free holotomography, in combination with cell and nuclear morphological features, as sensitive biophysical indicators of microglial senescence and phagocytic capacity. Microglial senescence was induced using optimized hydrogen peroxide (H_2_O_2_) treatment and validated through p21 and pRPS6 expression, and Raman spectroscopic signatures. Subsequently, phagocytosis assays were conducted following Aβ treatment. Results showed that dry mass showed a strong correlation with phagocytic decline in senescent cells. Senescent microglia treated with Aβ exhibited significantly lower dry mass and smaller cell size, but larger nuclear size, compared with Aβ-treated control microglia. These findings highlight dry mass as a robust, non-invasive biophysical indicator of microglial senescence and associated phagocytic function.

## Introduction

Microglia, the resident immune cells of the central nervous system (CNS), play a crucial role in maintaining CNS homeostasis by responding to injury and infection and clearing cellular debris through phagocytosis (Greenwood and Brown 2021; Malvaso et al. 2023; Wood 2024). Recent studies have identified disease-associated microglia (DAM) with distinct transcriptional profiles in the brains of elderly individuals, patients with neurodegenerative disorders, and relevant mouse models, implicating DAM in the pathogenesis of age-related neural diseases (Colonna and Butovsky 2017; Prinz et al. 2021). Therefore, microglial dysfunction may be a key contributor to CNS degeneration (Pathipati et al. 2013; Hansen et al. 2018; Greenwood and Brown 2021; Prinz et al. 2021; An et al. 2022; Pallarés-Moratalla and Bergers 2024; Wood 2024).

Microglial senescence is a state of irreversible cell cycle arrest in which the cells remain metabolically active (Rim et al. 2024; Singam et al. 2024). This state is marked by several characteristic changes, including altered morphology, vacuolization, and the secretion of senescence-associated secretory phenotype (SASP) factors (Ng et al. 2023; Malvaso et al. 2023). However, a universal marker for cellular senescence remains elusive, as the characteristics of senescent cells vary considerably depending on both cell type and the nature of the inducing stressor (Park et al. 2024; Kim et al. 2025). As a result, there is a continued need to identify additional senescence markers or to employ a combination of multiple markers to more accurately assess the degree of senescence (Severino et al. 2000; YANG and HU 2005; Liendl et al. 2020; Ogrodnik 2021; Safwan-Zaiter et al. 2022). In a recent study, we found that mechanical alterations during senescence, specifically changes in organelle transport dynamics in hydrogen peroxide (H_2_O_2_)-induced microglial cells, may serve as a potential marker of senescence (Singam et al. 2025). In particular, we observed that key transport parameters of lipid droplets, including velocity, mean squared displacement (MSD), and mean directional change, were significantly altered during cellular senescence.

One of the defining features of senescent microglia is impaired phagocytic function, which compromises their ability to clear cellular debris (Wood 2024). In particular, the phagocytosis of amyloid-β (Aβ), a peptide fragment that aggregates into extracellular plaques characteristic of Alzheimer’s disease, is markedly reduced in senescent microglia, potentially exacerbating disease progression (Gouwens et al. 2016; An et al. 2022). Despite its importance, however, there remains a lack of quantitative biophysical metrics to assess microglial phagocytic capacity during senescence.

In this study, we demonstrate for the first time that cellular dry mass, correlated with cell and nuclear size, all of which are non-invasively quantified via holotomography, can serve as a quantitative indicator of phagocytic function in senescent microglial cells. To induce senescence, microglial cells were treated with an optimized concentration of H_2_O_2_, and cellular senescence was confirmed using established senescent markers, including senescence-associated β-galactosidase (SA-β-gal) and p21 through immunostaining, followed by molecular signatures by Raman spectroscopy. Phagocytosis assays were then conducted by treating the cells with Aβ. Subsequently, time-lapse holotomographic imaging was performed to obtain RI (Refractive Index) distributions. Based on the measured RI, cellular dry mass and morphological properties were quantitatively assessed and compared between healthy control and senescent cells to evaluate correlations among dry mass, cell and nuclear morphology, microglial senescence, and phagocytic function.

## Materials and method

### Cell culture and reagents

Human microglial cells (HMC-3, CRL-3304, ATCC) were cultured in minimum essential medium (MEM) supplemented with 10% fetal bovine serum (FBS) and 1% penicillin-streptomycin at 37°C in a humidified 5% CO_2_ atmosphere. Cells were harvested at 70–80% confluency for up to 20 passages using 0.25% trypsin-EDTA (Gibco, USA).

### Induction of cellular senescence and quantification of intracellular reactive oxygen species (ROS)

Intracellular reactive oxygen species (ROS) production in HMC-3 cells was monitored using the 2’,7’-dichlorodihydrofluorescein diacetate (DCFDA) assay (Kato 2025). A 5 mM stock solution of DCFDA was prepared by dissolving the compound in anhydrous dimethyl sulfoxide (DMSO) and subsequently diluted to a working solution of 5 μM in serum-free, phenol-red-free medium for experiments. Approximately 50,000 cells per well were seeded in a 24-well plate and allowed to adhere and grow overnight. The following day, cells were treated with 50, 75, or 100 μM H_2_O_2_ for 1 hour to induce cellular senescence, followed by incubation under standard culture conditions for an additional 23 hours (Song et al. 2014; Gerasymchuk et al. 2022). After incubation, cells were washed with DPBS and incubated with 200 μl of DCFDA working solution in the dark at 37°C for 1 hour. Cells were then washed again with DPBS and imaged using a confocal microscope (Nikon A1Rsi, Nikon, Japan) at 20× magnification under the 488 nm (green) filter.

### Immunofluorescence staining

SA-β-gal activity was assessed using the CellEvent^™^ Senescence Green Detection Kit (Invitrogen, Cat# C10851, USA) (Singam et al. 2025). Briefly, after a 1-hour H_2_O_2_ treatment followed by a 23-hour incubation in medium, microglial cells were washed with buffer and fixed with 4% paraformaldehyde for 12 minutes at room temperature. The fixed cells were then washed with buffer, followed by treatment with 0.1% Triton X-100 (ThermoFisher Scientific, USA) and 1% BSA (Sigma-Aldrich, Cat# A9647, USA). The working solution (X-Gal) was prepared according to the manufacturer’s instructions and added to the wells. The plate was covered and incubated for 2 hours at 37°C in the dark without CO_2_. After incubation, the working solution was removed, and the wells were washed three times with buffer. For immunofluorescence staining of p21, HMC-3 cells were seeded and treated with varying concentrations of H_2_O_2_ as described above. Cells were fixed with 2% paraformaldehyde for 15 minutes, washed, and permeabilized with 0.3% Triton X-100 in PBS (PBS-T) for 10 minutes. After permeabilization, cells were incubated in blocking buffer containing 5% bovine serum albumin (BSA) and 0.3% Triton X-100. The primary antibody against p21 Waf1/Cip1 (rabbit, #2947, 1:500, Cell Signaling Technology, USA) was diluted in 1% BSA/PBS-T and incubated overnight at 4°C. The following day, cells were incubated with the secondary antibody, Alexa Fluor 594-conjugated anti-rabbit IgG (#8889, 1:2000, Cell Signaling Technology, USA), for 2 hours at room temperature in the dark. Similarly, cells were stained for phospho-S6 ribosomal protein (pRPS6, Ser235/236) (rabbit, #4858, 1:300, Cell Signaling Technology, USA), followed by incubation with the corresponding Alexa Fluor 594-conjugated secondary antibody, along with DAPI. Confocal images were acquired using a Leica Stellaris microscope (Wetzlar, Germany) at 40× magnification, and fluorescence intensity was quantified using ImageJ for subsequent statistical analysis.

### Raman spectroscopy

Cells were fixed onto crystalline quartz coverslips using the procedure described in the text and immersed in the PBS solution at room temperature for short-term storage during Raman measurement (a few hours) or in a refrigerator for long-term storage (overnight). We used the crystalline quartz coverslips to avoid background contributions to the Raman spectra. During Raman acquisitions, the coverslip was removed from the buffer and placed into the Raman system, and the buffer was allowed to evaporate prior to starting acquisitions. Total acquisition times were limited to less than 3 hours to prevent “drying out” of the cells prior to re-submerging the coverslip in the buffer solution. Raman spectra were acquired for 300 s per region and averaged nine times, spanning 600–1950 cm^−1^ across three spectral windows in nuclei from three control and four senescent cells. Raman scattering in cell nuclei was generated with a λ = 532 nm SpectraPhysics laser and collected in standard backscattering geometry using a home-built Raman spectroscopy system at the Nevada Extreme Conditions Laboratory (NEXCL). The laser beam was focused onto the sample, and the resulting Raman scattering was collected using an Olympus MPlan N 50× objective. The laser power incident on the sample was attenuated to prevent cell damage using ND optical filters; for the spectra displayed in this paper, the laser power at the focal point of the objective was set to approximately 1.2 mW. The full width at half maximum (FWHM) of the focal spot is approximately 1 × 1 μm^2^. Spectra were collected using a Princeton Instruments SP2750i spectrometer equipped with a PIXIS-100 CCD and 1800 g/mm grating, providing a wavenumber resolution of approximately 0.4 cm^− 1^. Following wavelength calibration using Hg-Ar gas, the system wavenumber scale was verified to within +/− 1 cm^−1^ of the 520 cm^−1^ Raman peak of crystalline silicon as reference.

### Preparation and phagocytosis assays of amyloid-beta (Aβ) fibrils

Human Aβ (1–42) fibrils (HiLexa Fluor^™^ 488-labeled, AnaSpec, AS-60479–01, USA) were prepared for microglial phagocytosis assays as previously described (Samentar et al. 2021). Briefly, 3 μL of Aβ films were diluted with 27 μL of 10 mM HCl to a final volume of 30 μL and vortexed for 10 minutes. The solution was then incubated for 24 hours with vigorous shaking to promote fibril formation. Immediately prior to use on day 2, the fibril stock was diluted to 100 nM by adding 990 μL of serum-free, phenol-red-free medium to 10 μL of the stock solution. Microglial cells pretreated with 50, 75, or 100 μM H_2_O_2_ were incubated with 100 nM Aβ fibrils for 30 minutes, followed by three washes with DPBS. Cells were then imaged using a confocal microscope (Nikon A1Rsi, Nikon, Japan) with excitation at 488 nm.

### Phagocytosis assays using IgG FITC beads

Phagocytosis assays were performed on microglial cells using IgG-coated, FITC-labeled nanobeads (Cayman Chemicals, 500290, USA; mean diameter = 100 nm) (Mysore et al. 2021). Briefly, microglial cells were seeded into 24-well plates and cultured overnight. Cells were then treated with 50, 75, or 100 μM H_2_O_2_ for 1 hour, followed by incubation under standard culture conditions for an additional 23 hours. After incubation, cells were washed with DPBS, and 200 μL of IgG-FITC beads (1:200 dilution) were added to each well for 2 hours to allow phagocytosis. Subsequently, cells were washed with Cayman buffer, stained with DAPI, washed three times with DPBS, and imaged using a fluorescence microscope (EVOS M5000, Invitrogen, USA) with excitation at 488 nm.

### Quantification of phagocytosis based on fluorescence intensity

To assess microglial phagocytic activity, the fluorescence intensity of internalized Aβ was quantified using image quantification techniques. Briefly, image pixels were converted to physical length scales, and fluorescence intensity within each cell was measured using ImageJ software. A total of 100 cells from three independent experiments were analyzed, and the data were averaged for healthy control cells and H_2_O_2_-induced senescent cells. A similar analysis was performed for the IgG-FITC beads. For these beads, fluorescence intensity was normalized to cell area (n = 25 cells) using ImageJ.

### Holotomography imaging and analysis

3D holotomographic imaging (3D Cell Explorer-fluo, Nanolive, Switzerland) was employed to analyze the morphology and intracellular composition of control and senescent microglial cells (Lopez-Osorio et al. 2022; Pérez-Montero et al. 2023; Lee et al. 2023; Kim et al. 2024a; Sbrana et al. 2024). This label-free technique uses RI tomography to generate high-resolution 3D reconstructions by quantifying RI variations across cellular structures (Pérez-Montero et al. 2023; Kim et al. 2024a; Sbrana et al. 2024; Singam et al. 2025). By exploiting the scattering and phase-shifting behavior of light as it passes through biological specimens, the phase delay that is proportional to the RI and sample thickness is captured via quantitative phase imaging. The holotomographic microscope operates with a low-energy 520 nm laser in an interferometric configuration, splitting the beam into an object beam that interacts with the sample and a reference beam. Their interference produces a hologram encoding spatial optical information. Holograms were acquired using a rotational scanning mirror, recorded by a CMOS camera, and computationally reconstructed into 3D RI maps using proprietary algorithms, allowing detailed visualization of subcellular components and material distribution. The system produces 3D z-stacks of 96 optical sections across a 30 μm depth. Built-in autofocus and motorized mirror stabilization ensure consistent image quality during extended acquisition.

Microglial cells were cultured in μ-Dish 35 mm plates (Ibidi GmbH, Germany) and imaged at 60× magnification with an air objective lens (NA = 0.8), providing sufficient resolution to delineate cellular boundaries and organelles such as nuclei, mitochondria, and lipid droplets. After bringing each cell into sharp focus, a 3D image was captured following calibration. To induce senescence, the culture medium was replaced with fresh medium containing 100 μM H_2_O_2_, and the same cells were re-imaged using identical acquisition settings. All imaging was performed in a controlled environment maintained at 37°C and 5% CO_2_ using an Okolab stage-top incubator. Quantitative analysis of cellular dry mass was conducted using the EVE Explorer software (Nanolive), which calculates dry mass from the integral of RI over the cellular volume or area, providing a biophysical measure of biomass content, as reported previously (Sandoz et al. 2019). Cell and nuclear area and perimeter were measured both using the EVE Explorer software (Nanolive) and ImageJ.

### Statistical analysis

Statistical analyses were performed using GraphPad Prism. Comparisons between two groups were conducted using a two-tailed unpaired Student’s t-test with Welch’s correction, while comparisons among four groups were analyzed using one-way ANOVA followed by Šídák’s post hoc test. Significance levels are indicated as follows: ns, not significant; *p < 0.05; **p < 0.01; ***p < 0.001; ****p < 0.0001. All data are presented as mean ± standard error of the mean (S.E.M.).

## Results

### Identification of senescence under varying H_2_O_2_ concentrations through molecular and compositional changes

**Figure 1:**
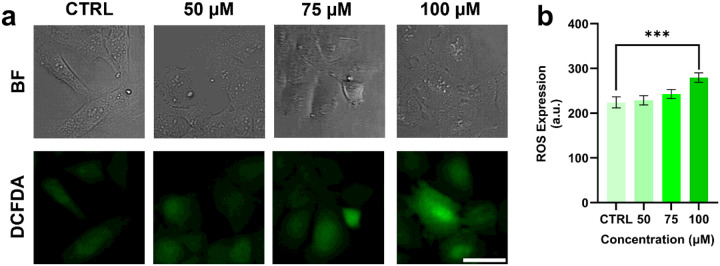
Qualitative and quantitative analysis of reactive oxygen species (ROS) levels in response to H_2_O_2_ treatment using immunofluorescence. (a) Fluorescence images (green) showing ROS accumulation in HMC-3 cells treated with increasing concentrations of H_2_O_2_ (50 μM, 75 μM, and 100 μM). Scale bar: 50 μm. (b) Quantification of ROS levels based on fluorescence intensity. Both qualitative and quantitative results demonstrate that ROS levels increase with higher concentrations of H_2_O_2_. BF: bright field. CTRL: control. n = 100 cells. ***p ≤ 0.001.

We first examined the effects of H_2_O_2_ on intracellular ROS levels, an indicator of oxidative stress, in microglial cells using the DCFDA fluorescent probe (Fig. 1a). Increasing concentrations of H_2_O_2_ resulted in progressively higher DCFDA fluorescence, reflecting enhanced ROS production. Notably, cells treated with 100 μM H_2_O_2_ exhibited significantly higher fluorescence intensity (279.67 ± 10.57 a.u.) than untreated control cells (224.27 ± 12.37 a.u.), corresponding to an approximate 25% increase (Fig. 1b). When microglial cells were exposed to increasing concentrations of H_2_O_2_, nuclear size showed a general upward trend (Fig. 4b), consistent with previous reports linking nuclear enlargement to senescence (Wei et al. 2025; Singam et al. 2025).

**Figure 2:**
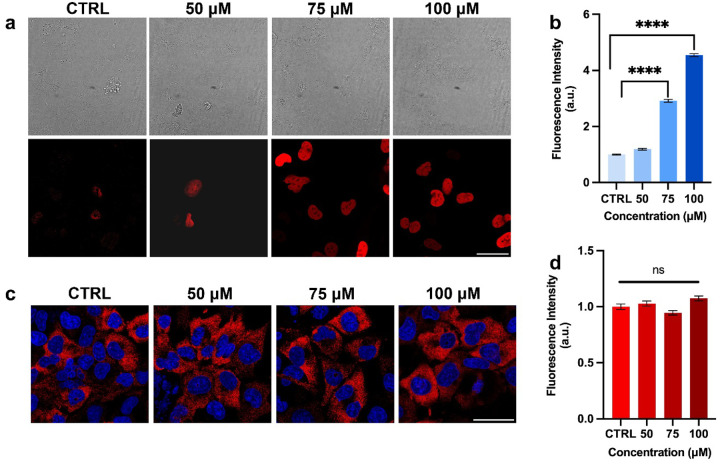
Effects of H2O2 concentrations on p21 and pRPS6 expression in microglia. (a) Brightfield and fluorescence images of p21. (b) Quantification of p21 fluorescence intensity. (c) Fluorescence images of pRPS6 (red) and nuclei (blue). (d) Quantification of pRPS6 fluorescence intensity. Higher concentrations of H2O2 lead to greater p21 expression while maintaining constant pRPS6 levels, indicating that the cells become senescent but remain metabolically active. Scale bar: 50 μm. BF: bright field; CTRL: control; n = 80 cells. ****p ≤ 0.0001; ns: not significant.

While both senescent and quiescent cells exhibit minimal growth and proliferation, senescent cells remain metabolically active and continue protein synthesis, whereas quiescent cells do not. To further distinguish cellular senescence from quiescence, we performed immunofluorescence staining for p21, a well-established senescence marker (Ben-Porath and Weinberg 2005; Campisi 2013), and phosphorylated RPS6 (pRPS6), a marker associated with protein synthesis and cell metabolism. As shown in Figs. 2a–b, p21 expression was low in healthy nuclei but increased markedly-approximately 3-fold and 4.5-fold-following treatment with 75 μM and 100 μM H_2_O_2_, respectively. In contrast, pRPS6 staining (Figs. 2c–d) showed relatively stable expression across control and H_2_O_2_-treated groups. These results clearly indicate that H_2_O_2_-treated cells are senescent rather than quiescent (Magnuson et al. 2012; Blagosklonny 2018; Alessio et al. 2021). In addition, in our previous work, we exhibited that SA-β-gal activity, another well-established senescent marker, increases with rising H_2_O_2_ concentrations, whereas concentrations above 100 μM lead to a sharp decline in viability, with survival falling below 50% (Singam et al. 2025). Based on these findings, and on the present observations of ROS accumulation (Fig. 1) and p21/pRPS6 induction (Fig. 2), we selected 100 μM H_2_O_2_ as the fixed and optimal concentration for subsequent biophysical analyses of cell and nuclear size, RI, and total dry mass using holotomography and biochemical analyses via Raman spectroscopy.

### Senescence-related biochemical changes via Raman spectroscopy

**Figure 3:**
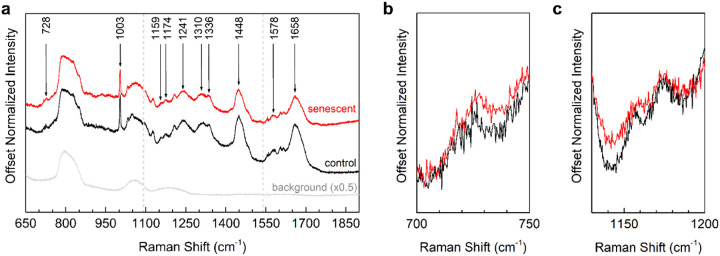
Raman spectroscopic analysis. (a) Comparison of average Raman spectra for control (black) and senescent (red) HMC-3 cells induced by 100 μM H_2_O_2_ and representative “off-cell” spectra (grey) showing spectral contributions of background. Cell spectra were normalized to the peak at 1003 cm^−1^; off-cell spectra were normalized to the highest intensity and scaled for visual comparison. Raman features of interest are denoted by arrows. Solid vertical lines at 1093 cm^−1^ and 1540 cm^−1^ mark where separate spectra have been “stitched” together. (b-c) Raman spectra of interest included the regions from 700–750 cm^−1^ (b) and 1130–1200 cm^−1^ (c), displaying significant differences between control and senescent cells.

To probe senescence-associated biochemical changes, we employed Raman spectroscopy, a label-free optical technique that probes molecular composition and structural features (Kim et al. 2025). Averaged Raman spectra of control and 100 μM H_2_O_2_-induced senescent nuclei (650–1900 cm^−1^) revealed several notable differences (Fig. 3a). The adenine-associated peak shifted from 726 cm^−1^ (CTRL) to 728 cm^−1^ (SEN) (Fig. 3b), a change consistent with senescence-associated DNA structural alterations (Shipp et al. 2017; Lopez-Lora et al. 2025). The peak at 1156 cm^−1^, attributed to C–C and C–N stretching of peptide bonds, presents a broadened band shifted to 1157 cm^−1^ for senescent nuclei, reflecting changes in protein conformation (Fig. 3c). Furthermore, senescent nuclei exhibited visibly reduced relative intensities at 1003, 1448, and 1658 cm^−1^, corresponding to nucleic acid and amide features (Ichimura et al. 2014; Tsikritsis et al. 2016).

### Phagocytosis of Aβ in microglial cells under varying H_2_O_2_ concentrations

**Figure 4:**
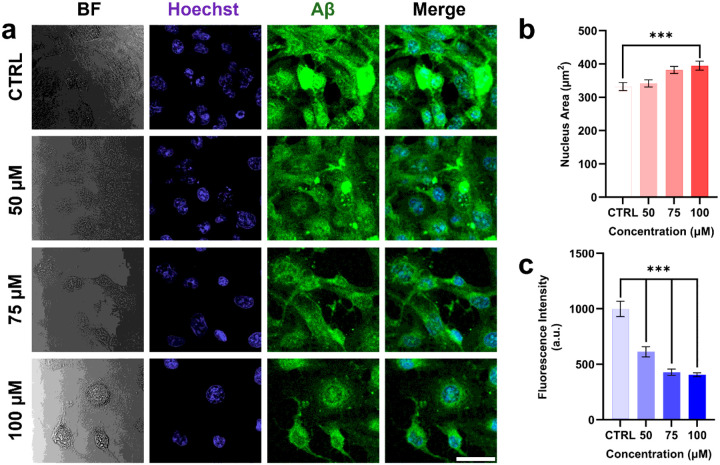
Phagocytosis assay of microglial cells under varying H_2_O_2_ concentrations. (a) Representative fluorescence images showing nuclei (purple) and internalized Aβ fibrils (green). Scale bar: 20 μm. (b) Quantification of nucleus area with varying H_2_O_2_ concentrations. (c) Quantification of phagocytic activity based on fluorescence intensity. Higher H_2_O_2_ concentrations result in reduced uptake of Aβ fibrils, indicating impaired phagocytic function. BF: bright field. CTRL: control. n = 100 cells. ***p ≤ 0.001.

To determine whether oxidative stress impacts microglial phagocytic ability, we quantified Aβ uptake using an immunofluorescence-based assay (Fig. 4a). Fluorescence analysis (Fig. 4c) showed that cells treated with 100 μM H_2_O_2_ exhibited markedly reduced Aβ internalization (405.83 ± 17.50 a.u.) relative to control cells (998.75 ± 69.81 a.u.) and cells treated with 50 μM (612.83 ± 45.71 a.u.) or 75 μM H_2_O_2_ (428.64 ± 28.27 a.u.).

To further validate this finding, we performed an independent phagocytosis assay using IgG-coated, FITC-labeled nanobeads. Quantification of bead uptake—calculated as the ratio of bead-occupied area to total cell area (**Fig. S1**)—similarly demonstrated that higher H_2_O_2_ concentrations impaired phagocytic function. The results showed that higher concentrations of H_2_O_2_ led to reduced bead uptake, indicating impaired phagocytic capacity in senescent microglia. Together, both Aβ fibril uptake and FITC-bead internalization assays consistently demonstrate that oxidative stress–induced senescence compromises microglial phagocytic function in a dose-dependent manner. These findings are consistent with previous reports showing that senescent or dysfunctional microglia exhibit reduced ability to engulf extracellular debris and Aβ aggregates, a decline closely associated with Alzheimer’s disease pathology (Thomas et al. 2022).

### Holotomographic analysis of biophysical alterations in senescent microglia

**Figure 5:**
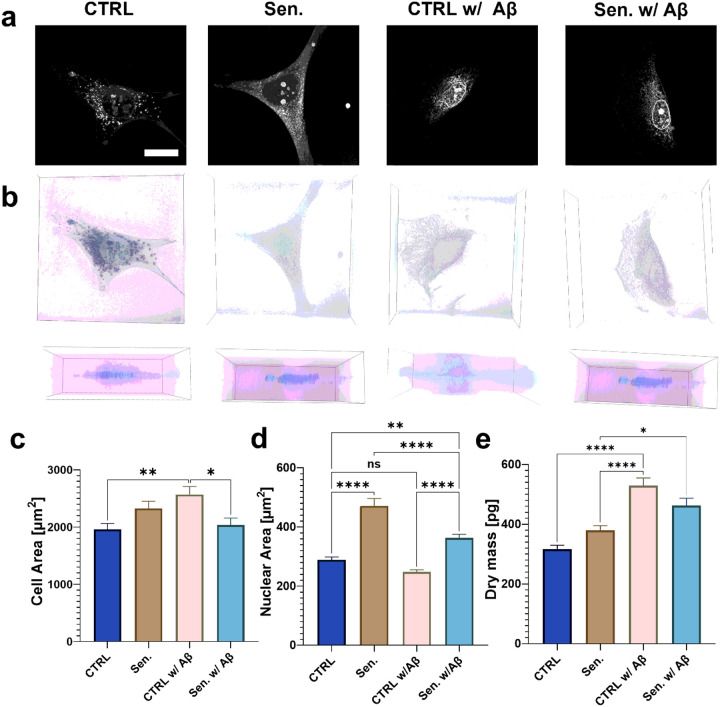
Quantification of microglial phagocytosis during cellular senescence. (a–b) Representative 2D a) and 3D (b) holotomographic images of control and senescent cells, with and without Aβ treatment. For (b), the top panel shows a top view, and the bottom panel shows a side view. Scale bar: 20 μm. (c–e) Cell area (c), nuclear area (d), and total dry mass per cell (e) in control and senescent cells, with and without Aβ. n = 50 cells. *p ≤ 0.05; **p ≤ 0.01; ****p ≤ 0.0001; ns: not significant.

To characterize senescence-associated biophysical changes, including dry mass, we used 3D holotomography to quantify RI–derived cell and nuclear area, and dry mass in microglial senescent cells treated with 100 μM H_2_O_2_ (Fig. 5). Holotomographic imaging enables label-free assessment of intracellular composition with high sensitivity, allowing direct correlation between senescence state and biophysical properties (Figs. 5a–b). Senescent cells exhibited enlarged cell bodies (2326 ± 124 μm^2^) and nuclei (470.6 ± 25.50 μm^2^), compared with controls (1959 ± 104.3 μm^2^) for cell body (Fig. 5c) and (288.4 ± 10.36 μm^2^) for nuclei (Fig. 5d), respectively. The increase in cell body size may be attributable to cytoplasmic accumulation of biomolecular cargo such as lipofuscin granules, glycogen aggregates, and stress granules, which reflects the well-established hypertrophic morphology characteristic of cellular senescence. For the nucleus, elevated senescence-associated heterochromatin foci (SAHF) can be a primary reason for the increase in nuclear size, including both cell area (Fig. 5c) and perimeter (**Fig. S2a**).

Next, we assessed the effect of Aβ exposure on these biophysical parameters. Overall, both Aβ-treated control and senescent cell groups exhibit markedly higher dry mass, mean RI intensity (**Fig. S2b**), and dry mass density (**Fig. S2c**) than their non-treated counterparts. Interestingly, while control cells exhibited increased dry mass (528.9 ± 26 pg) and cell area (2568 ± 143.4 μm^2^) after Aβ uptake, while maintaining similar size (247.8 ± 7.391 μm^2^) in the nucleus (Figs. 5c–e)—consistent with active phagocytosis—senescent cells failed to show a comparable increase. In fact, senescent microglia exposed to Aβ displayed reduced cell area (2035 ± 123 μm^2^), nuclear area (363 ± 12.25 μm^2^), and lower dry mass (462 ± 24.55 pg) relative to Aβ-treated control cells, further supporting impaired phagocytic function (Figs. 5c–e). The reduced dry mass (462 ± 24.55 pg) in senescent microglia exposed to Aβ, relative to control cells (528.9 ± 26 pg), reflects their diminished ability to internalize extracellular material. In contrast, the elevated dry mass (379 ± 15.87 pg) observed in untreated senescent cells, compared with untreated controls (316.2 ± 13.69 pg), results from the intracellular accumulation of senescence-associated deposits, lipids, and proteins. Collectively, these results demonstrate that quantitative metrics derived from holotomography—cell and nuclear area, and total dry mass—serve as sensitive indicators of both senescence progression and microglial phagocytic competence.

## Discussion

**Figure 6:**
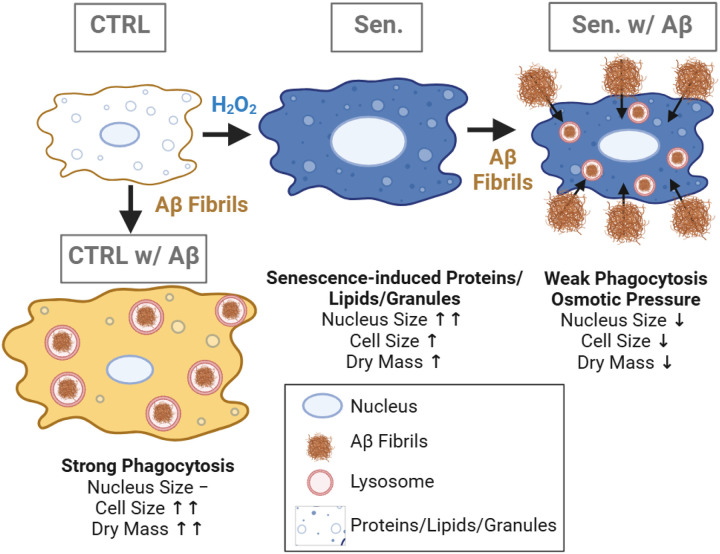
Graphical schematic of the proposed mechanisms underlying senescence- and Aβ-induced morphological and compositional changes. When healthy cells undergo senescence, cells and nuclei are enlarged with an increase in dry mass. Healthy control cells exhibit strong phagocytosis of Aβ, which is accompanied by a marked increase in cell area and dry mass, while senescent cells show weak phagocytosis but undergo high osmotic pressure, leading to reduced cell and nucleus size and dry mass.

In this study, holotomographic imaging was employed to examine alterations in dry mass in control and senescent microglial cells during Aβ phagocytosis. Consistent with previous reports (González-Gualda et al. 2021; Bresci et al. 2023), senescent cells exhibited a higher dry mass than control cells. This increase likely reflects the accumulation of intracellular deposits characteristic of senescence, including lipofuscin-containing lysosomes, glycogen particles, stress granules, lipid droplets, SAHFs, and other cytoplasmic debris, as reported previously (Kurz et al. 2000; Dodig et al. 2019; Csekes and Račková 2021; Huang et al. 2022; Bresci et al. 2023). With respect to Aβ phagocytosis, control microglia showed a marked increase in total dry mass upon Aβ exposure, whereas senescent cells exhibited only modest increases. This attenuated response is aligned with impaired phagocytic function in senescent microglia, resulting in reduced Aβ uptake. Consequently, the difference in dry mass between Aβ-treated and untreated conditions was substantially smaller in senescent cells than in controls. Together, these observations indicate that, while senescence intrinsically elevates dry mass, Aβ-induced phagocytic activity is better reflected by the magnitude of dry-mass change (Fig. 6).

We further quantified cellular and nuclear area to explore its relationship with dry mass. As expected, senescent cells displayed a significantly larger cellular and nuclear area than controls, consistent with well-established morphological hallmarks of cellular senescence. This enlargement may be driven by cytoskeletal remodeling, such as increased stress fibers and enhanced F-actin organization in cells and SAHFs in nuclei, as well as the accumulation of proteins, lipids, and dysfunctional organelles resulting from impaired autophagy (Debacq-Chainiaux et al. 2009; Aird and Zhang 2013; Campisi 2013; Gerasymchuk et al. 2022). Of note, control microglia exposed to Aβ showed pronounced cell enlargement accompanied by lamellipodial extensions (Figs. 5a–b), in line with previous observations (Lee and Landreth 2010; Kim et al. 2024b), possibly reflecting the increased volume and burden of internalized Aβ. In contrast, senescent microglia treated with Aβ exhibited a paradoxical reduction in cellular and nuclear area, potentially due to the combined effects of their limited phagocytic capacity and the osmotic or compressive effects of extracellular Aβ that they fail to internalize.

A key strength of this work is the integration of holotomography, a label-free, quantitative imaging modality, with functional Aβ phagocytosis assays. This approach enables direct assessment of phagocytic capacity by leveraging the clear correlation between Aβ uptake and increases in dry mass. It also allows discrimination between senescence-driven and phagocytosis-driven changes in intracellular content, providing a more mechanistic understanding than fluorescence intensity–based Aβ uptake alone. However, several limitations should be acknowledged. Because holotomography measures RI–derived dry mass, it cannot resolve molecular composition, limiting the ability to distinguish between lipids, proteins, nucleic acids, and other biomolecules underlying the observed changes. Complementary biochemical analyses, such as Raman spectroscopy, as implemented in this study, are necessary to identify molecular drivers of mass accumulation. Additionally, this analysis does not explicitly account for changes in cell shape or volume, both of which are key biophysical features of senescent cells and may influence dry-mass measurements. Future studies that integrate holotomographic dry-mass quantification with detailed morphological, biochemical, and functional assessments will be essential for fully delineating the biophysical underpinnings of microglial senescence.

## Conclusions

In this study, we examined biophysical changes associated with microglial senescence during Aβ phagocytosis by quantifying cellular dry mass as well as cell and nuclear size. We found that H_2_O_2_-induced senescence impairs microglial phagocytic function, as reflected by reduced uptake of fluorescent beads and Aβ. Senescent microglia also exhibit hypertrophy, with increased cell and nuclear area and dry mass in the absence of Aβ, due to elevated accumulation of deposits and debris. Interestingly, exposure to Aβ reduces both cell area and dry mass in senescent cells, highlighting their diminished phagocytic capacity. These results suggest that quantitative biophysical parameters, such as dry mass and cell size, can serve as reliable, non-invasive indicators of cellular senescence and microglial functional status, offering a potential method to assess their ability to clear pathological aggregates.

## Supplementary Material

Supplementary Files

This is a list of supplementary files associated with this preprint. Click to download.

- ManuscriptSupplement.docx

## Data Availability

The raw datasets generated during this study are available from the corresponding author upon reasonable request.
